# Glyphosate and glyphosate-based herbicides (GBHs) induce phenotypic imipenem resistance in *Pseudomonas* *aeruginosa*

**DOI:** 10.1038/s41598-022-23117-9

**Published:** 2022-10-29

**Authors:** Judit Háhn, Balázs Kriszt, Gergő Tóth, Dongze Jiang, Márton Fekete, István Szabó, Balázs Göbölös, Béla Urbányi, Sándor Szoboszlay, Edit Kaszab

**Affiliations:** 1 Department of Environmental Safety, Institute of Aquaculture and Environmental Safety, Hungarian University of Agriculture and Life Sciences, Gödöllő, Hungary; 2 Department of Environmental Toxicology, Institute of Aquaculture and Environmental Safety, Hungarian University of Agriculture and Life Sciences, Gödöllő, Hungary; 3 Department of Aquaculture, Institute of Aquaculture and Environmental Safety, Hungarian University of Agriculture and Life Sciences, Gödöllő, Hungary

**Keywords:** Applied microbiology, Environmental impact, Outcomes research

## Abstract

GBHs are the most widely used herbicides for weed control worldwide that potentially affect microorganisms, but the role of their sublethal exposure in the development of antibiotic resistance of *Pseudomonas* *aeruginosa* is still not fully investigated. Here, the effects of glyphosate acid (GLY), five glyphosate-based herbicides (GBHs), and POE(15), a formerly used co-formulant, on susceptibility to imipenem, a potent carbapenem-type antibiotic, in one clinical and four non-clinical environmental *P.* *aeruginosa* isolates were studied. Both pre-exposure in broth culture and co-exposure in solid media of the examined *P.* *aeruginosa* strains with 0.5% GBHs resulted in a decreased susceptibility to imipenem, while other carbapenems (doripenem and meropenem) retained their effectiveness. Additionally, the microdilution chequerboard method was used to examine additive/antagonistic/synergistic effects between GLY/POE(15)/GBHs and imipenem by determining the fractional inhibitory concentration (FIC) indexes. Based on the FIC index values, glyphosate acid and Total demonstrated a potent antagonistic effect in all *P.* *aeruginosa* strains. Dominator Extra 608 SL and Fozat 480 reduced the activity of imipenem in only one strain (ATCC10145), while POE(15) and three other GBHs did not have any effect on susceptibility to imipenem. Considering the simultaneous presence of GBHs and imipenem in various environmental niches, the detected interactions between these chemicals may affect microbial communities. The mechanisms of the glyphosate and GBH-induced imipenem resistance in *P.* *aeruginosa* are yet to be investigated.

## Introduction

Glyphosate acid (GLY), usually formulated in form of salts^1^, is the most widely used active ingredient in glyphosate-based herbicides (GBH) with an annual volume of 700,000 tons^2^. GLY interrupts the metabolic pathway of shikimic acid, the precursor for the biosynthesis of aromatic amino acids in plants, by inhibiting the enzyme that catalyzes the formation of its central intermediate, 5-enolpyruvylshikimate-3-phosphate synthase (EPSPS)^3^.

GLY and GBH use has been associated with adverse effects (cytotoxicity, carcinogenicity, teratogenicity, endocrine disruption, metabolic alterations) below the environmentally relevant concentrations or the recommended agricultural/horticultural usage doses^4–6^. Still, numerous GBHs are available on the worldwide pesticide market^7^ that may affect non-target organisms in aquatic-, and terrestrial ecosystems or threaten human health.

The shikimate pathway is present not only in the plants, but in bacteria and fungi as well, therefore, GLY can be considered as an antimicrobial agent^4^. GLY sensitivity partially depends on the class of EPSPS enzyme (class I, or class II) harbored by the microorganism: some *Proteobacteria* harboring the Class I EPSPS variant (*E.* *coli*, *Salmonella,* and species of *Pseudomonas*) may have higher GLY sensitivity^8^. Novel studies demonstrate the adverse effects of GLY and GBHs on environmental communities leading to changes in freshwater microbial communities^9^, influencing their activities in biogeochemistry cycles^10^, disturbing earthworms' gut microbiome^11^, and inducing microbiota dysbiosis in humans^12,13^ and in male Sprague–Dawley rats^14^. When it comes to assessing the influence of GLY, many studies focused on changes in soil and aquatic microbial community^15–21^ and verified that various microorganisms (especially bacteria) can use GLY as a sole source of phosphorus, carbon and nitrogen^22^.

Antibiotic resistance is a global, emerging problem that threatens human and animal health and raises environmental health concerns^23^ defined as an inherited ability of microorganisms to grow at high concentrations of an antibiotic, irrespective of the duration of treatment^24^. Over the last decades, the evolution of antibiotic resistance in pathogenic microorganisms correlated not only with the excessive antibiotic consumption but with the usage of biocides and commercial formulations of pesticides (e.g., 2,4-dichlorophenoxyacetic acid, dicamba, GLY)^25–27^. The association between antibiotic resistance of microorganisms and their GLY resistance was suggested by several authors^28,29^ and under the exposure to GLY isopropylamine (IPA) salt and Roundup, minimum inhibitory concentrations (MICs) of antibiotics in *Escherichia* *coli* and *Salmonella* *enterica* serovar Typhimurium isolates altered leading to antibiotic resistance phenotypes^26^. Exposure to sublethal concentrations of GLY may affect antibiotic susceptibility in *E.* *coli* and *S.* *enterica* as well^30^. Moreover, GLY can promote horizontal plasmid-mediated conjugative gene transfer of antibiotic resistance genes (ARGs) at 10 and 20 mg/L concentrations due to the increased cell membrane permeability in intragenic (*E.* *coli*) and intergenic (from *E.* *coli* to *Bacillus* *oleronius*) species^31^.

The correlation between the emergence of antibiotic-resistant bacteria and worldwide GLY use was suggested by several authors^4,27^. However, there is still a lack of knowledge about the effects of GLY and GBH exposure on the antibiotic resistance of *Pseudomonas* *aeruginosa,* a bacterium often found in locations associated with human activity^32^. *P.* *aeruginosa* is a frequent healthcare-associated pathogen with an outstanding capacity for antimicrobial resistance^33^, leading to the highest fatality rate of all opportunistic pathogen Gram-negative bacteria^34^. Due to their exceptional metabolic plasticity, *P.* *aeruginosa* strains are detectable in diverse ecological niches^35^ and can dominate contaminated sites^36^ with drug resistance, virulence, and biofilm-forming ability similar to their clinical counterparts^37–39^. *P.* *aeruginosa* can tolerate^27^ and degrade^40^ GLY, but the effects of sublethal exposure on its antibiotic resistance profile have not been investigated. To fill this gap, the initial aim of our research was to expose environmental and clinical isolates of opportunistic pathogen *P.* *aeruginosa* to sublethal concentrations of GLY and commercially available GBHs and to evaluate the induced alterations in the phenotypically detectable antibiotic resistance profiles. Based on the initial findings, our additional aim was to reveal the types of relationship (antagonism, synergism) between five chosen GBH formulations with different GLY salts and the formerly used co-formulant POE(15) (polyoxyethylene (15) tallow amine) with imipenem, a potent, carbapenem-type cell wall synthesis inhibitor^41^.

## Materials and methods

### Microorganisms

The examined five *P.* *aeruginosa* strains were chosen to represent various clinical and environmental sources (Table 1). Type strains were obtained from the National Collection of Agricultural and Industrial Microorganisms (NCAIM), Hungary, environmental strains were chosen from the strain collection of the Department of Environmental Safety (MATE), Hungary. All examined strains represented antibiotic sensitive phenotypes (Table S1). Species level identification performed with the PCR targeted the 16S rDNA variable regions V2 and V8 as it was described previously^42^.

**Table 1:** *P.* *aeruginosa* strains used in this study.

Strain	Species	Source
HF234 (this study)	*Pseudomonas* *aeruginosa*	Surface water, Hungary
P66^37^	*Pseudomonas* *aeruginosa*	Hydrocarbon contaminated groundwater, Hungary
ATCC 27853^43^	*Pseudomonas* *aeruginosa*	Clinical (isolated from blood)
ATCC 10145^44^	*Pseudomonas* *aeruginosa*	Type strain, unknown source
ATCC 15442^45^	*Pseudomonas* *aeruginosa*	Water bottle in animal room

### Glyphosate, POE(15) and GBH formulations

The stock solution of GLY [(N-(phosphonomethyl) glycine) with chemical formula: C_3_H_8_NO_5_P, CAS 1071-83-6] was prepared from Pestanal analytical standard (Merck Ltd., Germany). POE(15), with an average of 15 ethylene oxide groups (chemical formula: R-N(CH_2_CH_2_O)_m_-H (CH_2_CH_2_O)_n_-H, CAS 61791-26-2) with 100% purity was purchased from Greyhound Chromatography and Allied Chemicals. The examined commercially available GBHs, their declared GLY concentrations, and co-formulants are summarized in Table 2.

**Table 2:** Chemical properties of the examined water soluble concentrates of glyphosate-based herbicides (GBHs) examined in this study based on their Material Safety Data Sheets (MSDS).

Formulation	Declared glyphosate concentration (g/L)		Type of glyphosate salt	Co-formulant(s) % (w/w)	Expiration date of approval in Hungary
	Glyphosate acid (active substance)	Glyphosate salt			
Dominator Extra 608 SL	480	608	Dimethylamine (DMA) salt	d-Glucopyranose, oligomers, decyl octyl glycosides (< 5%), disodium cocoamphodipropionate (< 5%) Methyl alcohol (< 1%)	31.12.2025
Fozat 480	360	480	Isopropylamine (IPA) salt	Non-declared	31.12.2023
Gladiator 480 SL	360	486	Isopropylamine (IPA) salt	Polyethoxylated (15) tallow amine [POE(15)] (13–18%)	30.11.2016
Roundup Mega	450	551	Potassium (P) salt	Ethoxylated ether alkylamine (7%)	31.12.2025
Total	360	486	Isopropylamine (IPA) salt	Non-declared (inert substance)	31.12.2025

### Preliminary screening assay

Preliminary experiments aimed to examine the effects of five commercially available GBHs (Table 2) on the growth and antibiotic resistance profile of a representative isolate of *P.* *aeruginosa* (HF234) originating from surface water, Hungary (Table 1). 45 mL Luria–Bertani (LB) medium (10.0 g tryptone, 5.0 g yeast extract, and 9.0 g NaCl in 1000 mL distilled water) was inoculated with 5.0 mL overnight bacterial suspension calibrated to an optical density of OD_600_ = 0.6 ± 0.02. The medium was supplemented with GBHs to reach a final GBH concentration of 0.5% (v/v), equivalent to 1.8–2.8 g/L GLY acid depending on the type of formulation. This concentration falls within the recommended dilution range of GBHs [0.2–3.5% (v/v)] for agricultural and household use and similar to those found in water after agricultural practices^46^. The experiment was performed in triplicates with an incubation period of 72 h at 28 °C on a horizontal shaker. During incubation, growth curves were determined based on regularly measured OD_600_ values to verify the sublethal effect of the chosen concentration. The antibiotic resistance assay was performed at the start (0 h) and endpoints (72 h) of the experiment using GBHs free Mueller–Hinton agar (Merck Ltd., Germany). Antibiotic resistance was examined with Liofilchem MIC test strips to determine Minimal Inhibitory Concentrations (MICs) according to the recommendations of the European Committee on Antimicrobial Susceptibility Testing (24 h incubation at 35 °C)^47^. The tested antibiotic agents represented six groups of antibiotics (Table S1). MIC differences between non-treated and pre-exposed cultures were analysed with a Two-way ANOVA multiple comparison (Tukey) test.

To evaluate the effect of co-exposure, preliminary assay was repeated with three GBHs (Roundup Mega, Dominator Extra 608 SL, Gladiator 480 SL) that significantly modified MICs of imipenem after 72 h pre-exposure. The overnight cultures of five environmental and clinical reference strains of *P.* *aeruginosa* (listed in Table 1) were involved in this setting. Bacterial suspensions were used without pre-exposition and were spread directly onto Mueller–Hinton agar containing 0.5% (v/v) GBHs. Antibiotic resistance test was performed as it was described above.

### Microplate dilution assay

Based on the results of the preliminary assays, the imipenem-resistance inducing effect of GBHs, GLY, and its formerly used co-formulant POE(15) was examined further by the microdilution chequerboard method ^48^ with slight modifications. Assays were performed on 96-well, clear, U-shaped PS microplates (Greiner Bio-One GmbH, Austria) using two-fold dilution series of 2 g/L imipenem (CAS 74431-23-5, Supelco, Sigma-Aldrich Ltd.), 10.0 g/L GLY, 5 g/L POE(15) and 50% (v/v) GBH stock solutions prepared in sterile distilled water. Test materials were co-added to the x-, and y-axes across the plate to reach 0–64 mg/L imipenem (x-axis) and 0–800 mg/L GLY, 0–4 mg/L POE(15), 0–4% (v/v) GBH (y-axis) final concentrations, respectively. Controls containing only GLY/POE/GBH or imipenem have been carried out. Concentration ranges considered the water-solubility of the examined compounds.

50 µL of overnight *P.* *aeruginosa* bacterial suspensions with optical density of OD_600_ = 0.6 ± 0.02 were added to each well. The final volume was adjusted to 250 µL with LB broth medium. 200 µL LB supplemented with 50 µL bacterial suspension was used as negative control.

Assays were carried out in triplicates using freshly prepared solutions and bacterial suspensions. Plates were incubated at 28 °C and at a speed of 350 rpm in a microplate shaker thermostat (PST-60HL-4, BioSan, Latvia). Absorbance was measured by an ELx800 microplate reader at 550 nm at the beginning of the incubation (0 h) and after 24 h of exposure.

### Data analysis

Statistical data analysis and visualization were performed using GraphPad Prism 7 software, version 7.00 (GraphPad Software Inc., San Diego, USA). Replicate measurements of microplate assays were averaged prior to statistical evaluation. Absorbance data were expressed in mean values for heatmap visualization. To examine the additive/antagonistic/synergistic effects between imipenem and the examined glyphosate related chemicals, fractional inhibitory concentration index (FICI) was calculated using the following mathematical expression^49^:

$$\begin{gathered} FIC_{A} = \, \frac{{MIC\left( {A \, in \, the \, presence \, of \, B} \right)}}{{MIC\left( {A \, alone} \right)}} \hfill \\ FIC_{B} = \, \frac{{MIC\left( {B \, in \, the \, presence \, of \, A} \right)}}{{MIC\left( {B \, alone} \right)}} \hfill \\ FIC_{A} + \, FIC_{B} = \, FICI \hfill \\ \end{gathered}$$

MIC is the minimal inhibitory concentration; FIC is the fractional inhibitory concentration; FICI is the fractional inhibitory concentration index.

FIC index of < 0.5 indicates synergism, 0.5–4.0 means indifference, and ≥ 4.0 is considered as antagonism^50^.

To calculate the differences between GLY/POE(15)/GBHs and imipenem, the average absorbance values of the examined five strains under co-exposition and their respective imipenem control (samples containing bacterial suspension and imipenem) were compared. The analysis of variance was performed with a two-way ANOVA, followed by Dunnett’s multiple comparisons tests. Differences with p ≤ 0.05 were considered significant.

## Results

### Preliminary screening assay: pre-exposure with GBHs increases MIC of imipenem

**Figure 1:**
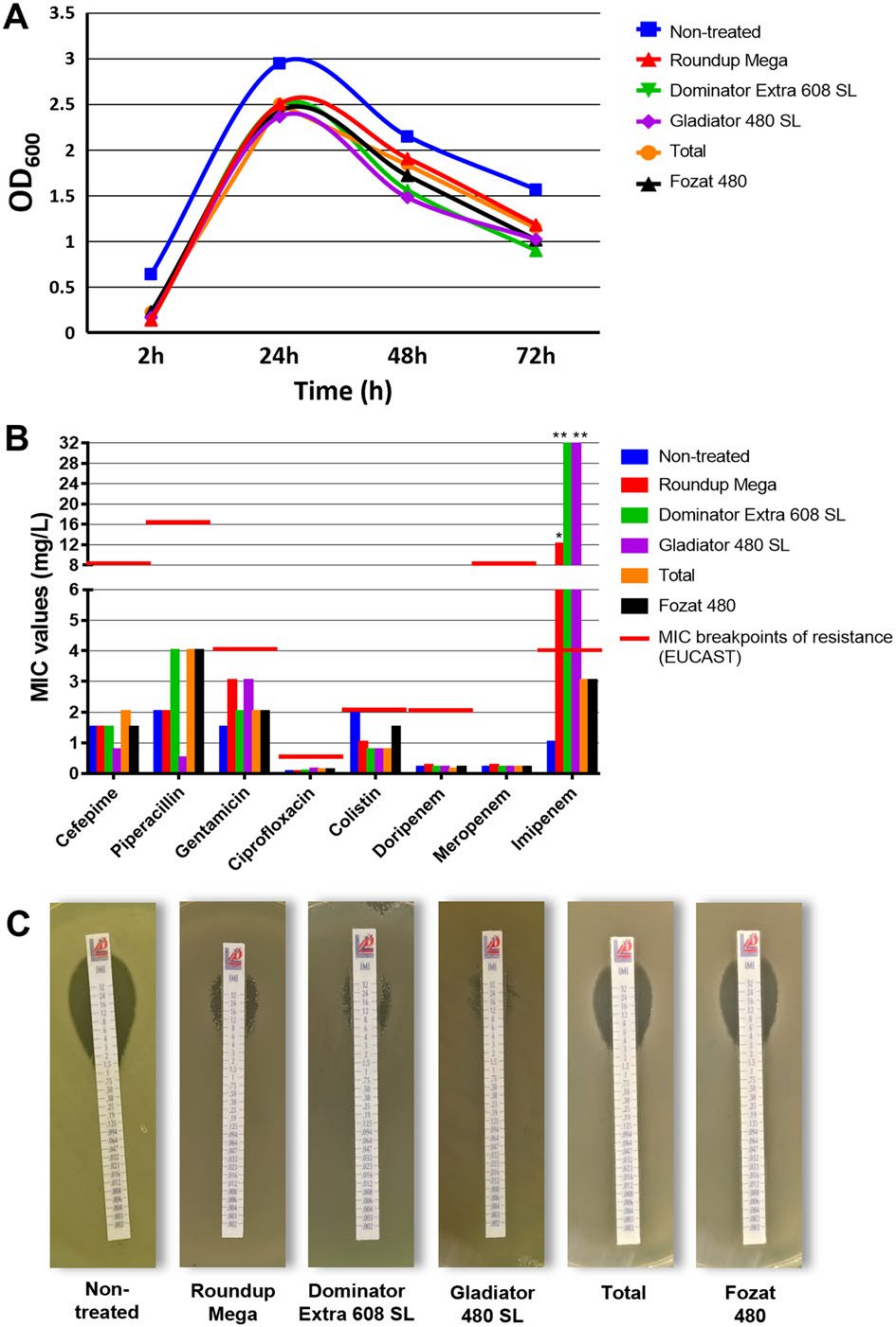
Preliminary liquid broth assay of *P.* *aeruginosa* HF234 with 0.5% (v/v) GBH pre-exposure (averaged values of three technical replicates). (**A**) Optical density (OD_600_) of broth cultures during the 72 h pre-exposure with GBHs. (**B**) MIC values of HF234 cultures after 72 h of pre-exposure with GBHs, plated on GBHs-free agar plates. (**C**) Inhibition zones of HF234 cultures after 72 h of pre-exposure with GBHs, plated on GBHs-free agar plates. *,**Significantly different from the non-treated strain (p = 0.0332–0.0023).

According to our preliminary screening results, the HF234 isolate of *P.* *aeruginosa* could tolerate 0.5% (v/v) of Roundup Mega, Dominator Extra 608 SL, Gladiator 480 SL, Total, and Fozat 480 without any significant inhibitory effect (Fig. 1A). The applied 0.5% GBH pre-exposure caused a 1.5–2 fold increase in MIC values of piperacillin and gentamicin, but the detected changes were not statistically significant (Fig. 1B). At the same time, MIC values of imipenem showed a 2–32-fold increase (Fig. 1B,C), while the other examined carbapenems (meropenem and doripenem) retained their effectiveness. According to Tukey’s multiple comparison test, Dominator Extra 608 SL Gladiator 480 SL and Roundup Mega pre-exposure induced a significantly higher level of resistance against imipenem.

### Preliminary screening assay: co-exposure with GBHs increases MIC of imipenem

The repeated imipenem assay, extended to five *P.* *aeruginosa* strains (listed in Table 1.), verified, that Dominator Extra 608 SL and Gladiator 480 SL induce similar levels of MIC alterations in the case of imipenem without pre-exposure if 0.5% (v/v) of GBHs are mixed directly into the Mueller–Hinton medium (MIC values ranged between 16 and 32 mg/L) (Table S2).

### Drug interactions in FIC assay: GBHs have an antagonistic effect on imipenem

**Figure 2:**
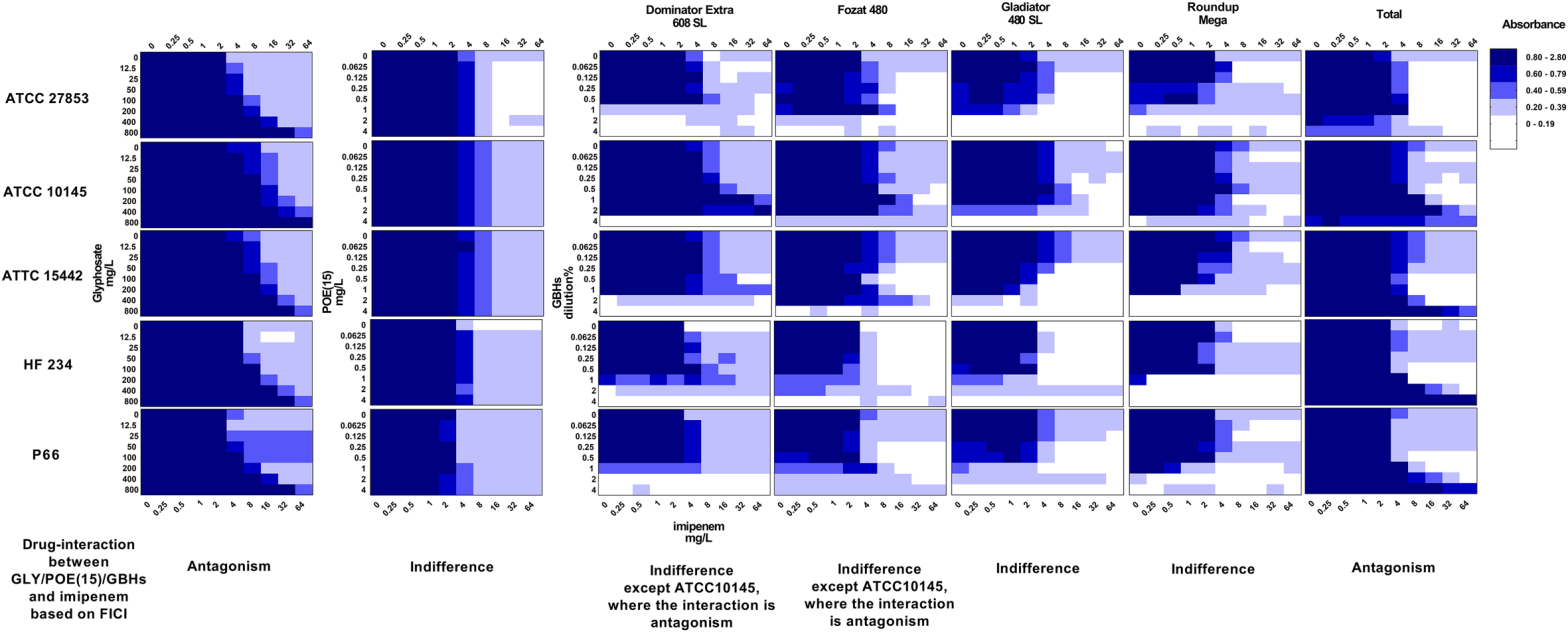
Heatmap of averaged absorbance values of the examined *P. aeruginosa* strains under co-exposure with different concentrations of GLY/POE(15)/GBHs and imipenem. X-axis: imipenem concentrations (0–64 mg/L), y-axis: concentrations of test materials (GLY: 0–800 mg/L, POE(15): 0–4 mg/L, GBHs: 0–4% (v/v)). Blue bars indicate relative fold growth ^51^. Drug-interactions between GLY/POE(15)/GBHs and imipenem were determined by the FIC index calculated as described in the methods (see details in Table 3).

Averaged values of absorbance determined by microplate chequerboard test of the examined five *P.* *aeruginosa* strains exposed to both imipenem and GLY/POE(15)/GBHs are summarized in Fig. 2.

**Figure 3:**
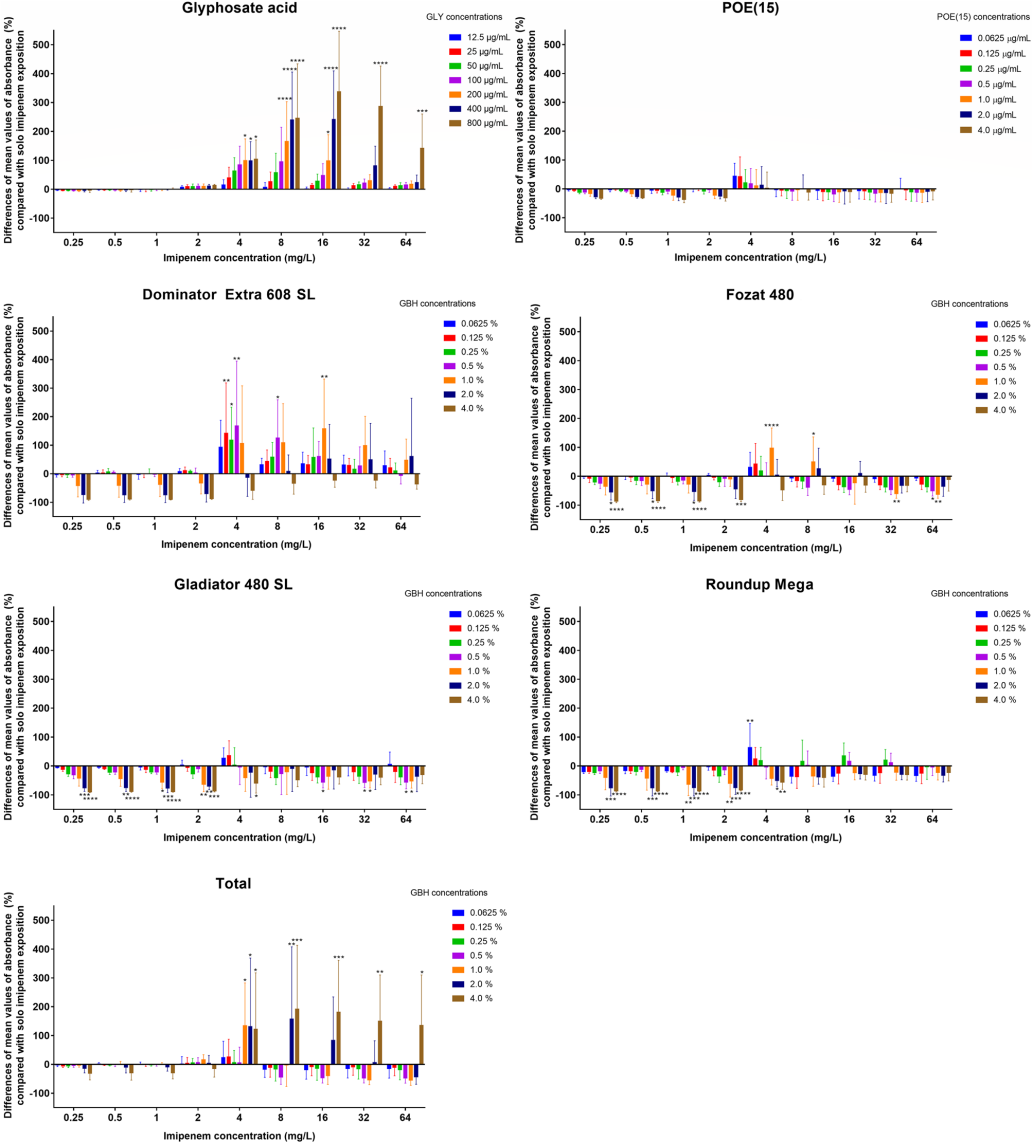
Summarized average differences of absorbance values (%) of all the examined five *P.* *aeruginosa* strains under co-exposition to imipenem with GLY, POE(15), and GBHs, compared with the absorbance values of solo imipenem exposition. X-axis: imipenem concentrations (0.25–64 mg/L) and seven concentrations of each test material (GLY: 12.5–800 mg/L, POE(15): 0.0625–4.0 mg/L, GBHs: 0.0625–4.0% (v/v)). Positive range—intensification; negative range—inhibition. Each bar represents the average absorbance value of five *P.* *aeruginosa* strains co-exposed to imipenem and test materials in three independent experiments. Two-way ANOVA and Dunnett’s multiple comparisons tests were used. *, **, *** and ****Significantly different from solo imipenem values (p < 0.0332, p < 0.0021, p < 0.0002 and p < 0.0001, respectively).

Results of FIC index determinations are available in Table 3. Summarized average percental differences in absorbance of *P.* *aeruginosa* strains after co-exposition to GLY/POE(15)/GBHs and imipenem compared to solo imipenem exposition are presented in Fig. 3.

**Table 3:** Fractional inhibitory concentration index (FICI) determination of *P.* *aeruginosa* strains co-exposed to imipenem and glyphosate related chemicals (glyphosate acid, POE(15), glyphosate-based herbicides).

	Imipenem (mg/L)					Imipenem (mg/L)					Imipenem (mg/L)					Imipenem (mg/L)					Imipenem (mg/L)					Imipenem (mg/L)					Imipenem (mg/L)				
	MIC_A_					MIC_A_					MIC_A_					MIC_A_					MIC_A_					MIC_A_					MIC_A_				
	Glyphosate acid (mg/L)					POE(15) (mg/L)					Dominator extra 608 SL, % (v/v)					Fozat 480, % (v/v)					Gladiator 480 SL, % (v/v)					Roundup mega, % (v/v)					Total, % (v/v)				
	MIC_B_					MIC_B_					MIC_B_					MIC_B_					MIC_B_					MIC_B_					MIC_B_				
	ATCC27853	ATCC10145	ATCC15442	HF234	P66	ATCC27853	ATCC10145	ATCC15442	HF234	P66	ATCC27853	ATCC10145	ATCC15442	HF234	P66	ATCC27853	ATCC10145	ATCC15442	HF234	P66	ATCC27853	ATCC10145	ATCC15442	HF234	P66	ATCC27853	ATCC10145	ATCC15442	HF234	P66	ATCC27853	ATCC10145	ATCC15442	HF234	P66
MIC_A_ alone	2	2	2	4	2	2	2	2	2	2	2	2	2	2	2	2	2	2	2	2	1	2	2	2	2	2	2	2	2	2	1	2	2	2	2
MIC_A_ (in the presence of B)	32	> 64	32	32	32	2	2	4	2	1	4	> 64	4	4	2	4	8	2	2	2	2	4	2	2	2	2	4	4	2	2	4	16	16	> 64	16
FIC_A_	16	32	16	8	16	1	1	2	1	0.5	2	32	2	2	1	2	4	1	1	1	2	2	1	1	1	1	2	2	1	1	4	8	8	32	8
MIC_B_ alone	> 800	> 800	> 800	> 800	> 800	> 4	> 4	> 4	> 4	> 4	0.5	2	1	0.5	0.5	0.5	2.0	2.0	0.5	0.25	0.125	1.0	1.0	0.25	0.125	0.125	2.0	1.0	0.5	1,0	2.0	2.0	> 4.0	> 4.0	> 4.0
MIC_B_ (in the presence of A)	> 800	> 800	> 800	> 800	> 800	> 4	> 4	> 4	> 4	> 4	0.0625	1.0	0.0625	0.0625	0.0625	0.0625	0.0625	0.0625	0.0625	0.0625	0.0625	0.0625	0.0625	0.0625	0.0625	0.0625	0.0625	0.0625	0.0625	0.0625	0.0625	0.0625	0.0625	4.0	0.0625
FIC_B_	1.0	1.0	1.0	1.0	1.0	1.0	1.0	1.0	1.0	1.0	0.125	0.5	0.0625	0.125	0.125	0.125	0.0313	0.0313	0.125	0.25	0.5	0.0625	0.0625	0.25	0.5	0.5	0.0313	0.0625	0.125	0.0625	0.0313	0.0313	0.0156	1.0	0.0156
FICI	17.0	33.0	17.0	9.0	17.0	2.0	2.0	3.0	2.0	1.5	2.125	32.5	2.062	2.125	1.125	2.125	4.0313	1.0313	1.125	1.25	2.5	2.0625	1.0625	1.25	1.5	1.5	2.0313	2.0625	1.125	1.0625	4.0313	8.0313	8.0156	33.0	8.0156
FICI (mean)	18.6					2.1					1.9 (mean of four strains)					1.9 (mean of four strains)					1.6					1.5					12.8				
FICI evaluation	Antagonism					Indifference					Indifference Except ATCC10145, where FICI = 32.5–antagonism					Indifference Except ATCC10145, where FICI = 4.03–antagonism					Indifference					Indifference					Antagonism				

FICI < 0.5—synergy; FICI = 0.5–4.0—indifference; FICI ≥ 4.0—antagonism.

According to our results, glyphosate acid was not toxic to *P.* *aeruginosa* in the examined range (12.5–800 mg/L), but it had a strong antagonistic effect (FICI_mean_ = 18.6) on imipenem and induced a concentration-dependent resistance in all strains; MIC of imipenem increased from 1–3 mg/L up to 32–64 mg/L, respectively. The strongest antagonistic effect was detected in the case of *P.* *aeruginosa* ATTC 10145: the viability of the culture in the simultaneous presence of 800 mg/L GLY and 64 mg/L imipenem was identical to that of the untreated control (Fig. 2).

POE(15) exposition had no concentration-dependent effect on the growth and imipenem sensitivity of the examined *P.* *aeruginosa* strains, but a slight increase in absorbance (up to 50%) was observed during co-exposition with 4 mg/L imipenem.

Of the five GBHs microplate assay verified that Total was not only non-toxic to the examined *P.* *aeruginosa* strains in the tested concentrations, but induced resistance towards imipenem: absorbance during co-exposition increased up to 192%, while MIC values of imipenem have significantly increased to 4–64 mg/L (Fig. 3, Table 3). FIC indexes verified the antagonistic effect between Total and imipenem (FICI was 12.8) in all strains. Dominator Extra 608 SL and Fozat 480 decreased susceptibility to imipenem in only strain ATCC10145 (FICIs were 32.5 and 4.03, respectively, indicating antagonism), while the interactions were indifferent in all the other strains (Table 3). The least pronounced effect of co-exposure was detected in the case of clinical reference strain ATTC 27853 with Total and P66 with Dominator Extra 608 SL, leading to only a slight increase (from 1 to 4 mg/L) in imipenem tolerance.

Gladiator 480 SL, Fozat 480, and Roundup Mega led to significant decreases in absorbance at higher [2–4% (v/v)] concentrations of GBHs, equivalent to 7.2–19.2 g/L glyphosate acid, verifying their cytotoxic effect on the examined strains (Figs. 2 and 3). At lower concentrations [0.0625–1.0% (v/v)], the above mentioned GBHs slightly increased imipenem tolerance compared to the solo exposure to the antibiotic, but the differences were significant in the case of Roundup Mega and Fozat 480 co-exposed with 4–8 mg/L imipenem only.

According to our results, the more pronounced antagonistic effect of Dominator Extra 608 SL and Total on imipenem can be related to their lower cytotoxicity to *P.* *aeruginosa*: their cytotoxicity was not significant even at higher concentrations [2.0–4.0% (v/v) equivalent to 7.2–14.4 g/L glyphosate acid]. Therefore, they could stimulate a more pronounced resistance to imipenem (up to 64 mg/L) in *P.* *aeruginosa.*

## Discussion

GLY, the most frequently used herbicide worldwide, is the focus of scientific and public discussions, especially in the European Union, where the renewing of its approval is approaching (currently approved in the EU until 15 December 2022). Recently, besides examining the acute and chronic biological effects (cytotoxicity, carcinogenicity, teratogenicity, endocrine disruption) of GLY^4–6^ and some GBHs^52,53^, the focus has been on the occurrence of antibiotic resistance evoked by GLY in soil and bacterioplankton communities^54,55^, *E.* *coli*^56^, and *Salmonella* strains^26,57^. At the same time, other pathogenic bacteria like *P.* *aeruginosa,* with a high capacity to tolerate and utilize GLY as the sole P or N source leading to its complete degradation within 96h^40^, have not been investigated from the antibiotic resistance point of view^27^.

We demonstrated for the first time that GLY acid significantly decreases susceptibility to imipenem in *P.* *aeruginosa* strains: with microplate chequerboard method we proved that all five examined isolates had a higher level of imipenem resistance in a concentration-dependent manner when they were exposed to GLY, but not to POE(15). Regarding GBHs, the examined four environmental and one clinical *P.* *aeruginosa* strain had a GBH tolerance up to 1–4% (v/v) (equivalent to 3.6–14.4 g/L GLY acid depending on the type of formulation), which are within the range of the commonly recommended doses for agricultural and horticultural use. The phenotypic imipenem resistance of GBH co-exposed *P.* *aeruginosa* strains was detectable at a concentration range of 4–64 mg/L, while the MIC breakpoint of imipenem resistance is 4 mg/L^58^. Out of five examined GBHs, the freely available, POE(15)-free, IPA salt containing Total caused pronounced increase in MICs of all strains during co-exposure with imipenem, while Fozat 480 and Dominator Extra 608 SL (containing IPA and DMA salt, respectively) decreased the efficiency of imipenem in only type strain ATCC 10145. Therefore, the type of GLY salt used for formulation was not a determining factor of the detected alteration in susceptibility to imipenem.

The nonheritable resistance to antibiotics in *E.* *coli* induced by salicylates and other chemotactic repellents^59^, the kanamycin resistance of *E.* *coli* in the presence of GLY^26^ and the kanamycin/ciprofloxacin resistance of *S.* Typhimurium induced by the exposure to Roundup^26^ were previously reported. The imipenem resistance of *P.* *aeruginosa* induced by GLY and GBHs has a similar pattern and rapidity: therefore, it may be activated by an efflux or permeability-related mechanism as it was suggested in the case of *E.* *coli*^26^, or by the activation of general mechanisms against potential stressors, which does not necessarily require a change in the specifically targeted structures^60^.

In clinical settings, carbapenem resistance of *P.* *aeruginosa* has been divided into three phenotypes: imipenem resistant-meropenem susceptible type I (IRMS), meropenem resistant-imipenem susceptible type II (MRIS), and imipenem resistant-meropenem resistant type III (IRMR). It has been described that IRMS is primarily caused by the numerous different mutations observed across various loops in the *oprD* porin leading to the porin down regulation^61^. At the same time, MRIS is due to the over-expression of the *mexAB-oprM* efflux operon in clinical *P.* *aeruginosa* strains, while strains harbouring plasmid-mediated carbapenemase genes usually belong to the type III (IRMR) group^61^. In our study, we have discovered phenotypic imipenem resistance in parallel with meropenem and doripenem susceptibility in both clinical and environmental *P.* *aeruginosa* strains after co-exposure to antibiotics and GBHs, therefore, we presume, that the detected changes can be related to the porin regulation of *oprD* as it was described in the case of IRMS type strains^61^ or to other general mechanisms against stressors^60^. The molecular mechanisms of the glyphosate and GBH-induced imipenem resistance in *P.* *aeruginosa* are yet to be investigated.

## Conclusions

Over the years, many studies have proved the harmful biological effects of GLY and GBHs or co-formulants used in GBHs. We report for the first time that GLY acid and freely available GBHs (containing a variety of co-formulants, but free of POE(15) induce significant, phenotypically detectable, discrepant imipenem resistance in clinical and environmental *P.* *aeruginosa* strains, while POE(15), the formerly used and banned co-formulant does not affect imipenem sensitivity. Considering the worldwide use of GLY and GBHs, and the simultaneous emergence of antibiotic-resistant bacteria in environmental matrices, the detected interactions between these chemicals may affect microbial communities and possess a potential environmental- and human health risk. Exploring the underlying mechanisms of this phenomenon is essential for further risk management.

## Data availability

The datasets generated during and/or analysed during the current study are available as Supplementary data. Further supplementary information is available from the corresponding author on reasonable request.

## Supplementary Information

Supplementary Tables.
