# Supplementary material for: Glyphosate and glyphosate-based herbicides (GBHs) induce phenotypic imipenem resistance in Pseudomonas aeruginosa
Source: Sci Rep. 2022 Oct 29;12:18258. doi: 10.1038/s41598-022-23117-9 (PMC9617868; doi:10.1038/s41598-022-23117-9)
Supplement: Supplementary file 1 — Supplementary Tables. [file 41598_2022_23117_MOESM1_ESM.docx]

**Supplementary information**

| **Table S1.** Antibiotics used in preliminary assay and their minimal inhibitory concentrations (MIC values) in *P. aeruginosa* strains without pre-exposure plated on GBHs-free Mueller-Hinton agar plates | | | | | | | | |
| --- | --- | --- | --- | --- | --- | --- | --- | --- |
| **Antibiotics** | **AwaRe classification*** | **Group** | **MIC breakpoints of resistance (R >)**** | **MIC values of *P. aeruginosa* strains (mg/L)** | | | | |
|  |  |  |  | **ATCC 27853** | **ATCC 10145** | **ATCC 15442** | **HF234** | **P66** |
| Cefepime | Watch | Cephalosporins | 8 | 8 | 8 | 3 | 1.5 | 2 |
| Ceftazidime | Watch | Cephalosporins | 8 | 1 | 3 | 2 | 2 | 1 |
| Piperacillin | Watch | Penicillins | 16 | 4 | 4 | 3 | 4 | 8 |
| Gentamicin | Access | Aminoglycosides | 4 | 1 | **12** | 1.5 | 2 | 0.75 |
| Ciprofloxacin | Watch | Fluoroquinolones | 0.5 | 0.75 | 0.38 | 0.09 | 0.064 | 0.13 |
| Colistin | Reserve | Polymyxins | 2 | **6** | **6** | **4** | **4** | **3** |
| Doripenem | Watch | Carbapenems | 2 | 1 | 1 | 0.5 | 0.19 | 1 |
| Meropenem | Watch | Carbapenems | 8 | 1.5 | 1.5 | 0.75 | 0.125 | 0.38 |
| Imipenem | Watch | Carbapenems | 4 | 2 | 3 | 0.75 | 1 | 1 |

*The 2019 WHO AWaRe classification of antibiotics for evaluation and monitoring of use. Geneva (2019). Available at: https://apps.who.int/iris/bitstream/handle/10665/327957/WHO-EMP-IAU-2019.11-eng.xlsx. (Accessed: 9th May 2022)

**EUCAST: Clinical breakpoints and dosing of antibiotics. Available at: https://www.eucast.org/clinical_breakpoints/. (Accessed: 9th May 2022)

MIC values above the EUCAST breakpoints of resistance are highlighted in bold

| **Table S2.** Minimal inhibitory concentrations (MIC values) in *P. aeruginosa* strains without pre-exposure plated on 0.5% (v/v) GBHs containing Mueller-Hinton plates | | | | | | |
| --- | --- | --- | --- | --- | --- | --- |
| **Antibiotic** | **GBH (0.5% v/v) content of the plates** | **MIC values of *P. aeruginosa* strains (mg/L)** | | | | |
|  |  | **ATCC 27853** | **ATCC 10145** | **ATCC 15442** | **HF234** | **P66** |
| Imipenem | Non-treated | 2 | 3 | 0.75 | 1 | 1 |
| Imipenem | Gladiator 480 SL | **>32** | **>32** | **>32** | **>32** | **>32** |
| Imipenem | Roundup Mega | 4 | 4 | **>32** | **12** | 4 |
| Imipenem | Dominator 608 SL | **>32** | **16** | **16** | **>32** | **>32** |

MIC values above the EUCAST breakpoints of resistance (4 mg/L) are highlighted in bold
